# Characterization of human norovirus binding to gut-associated bacterial ligands

**DOI:** 10.1186/s13104-019-4669-2

**Published:** 2019-09-23

**Authors:** Erin A. Almand, Matthew D. Moore, Lee-Ann Jaykus

**Affiliations:** 1 Department of Plant and Microbial Biology, North Carolina State University, Raleigh, NC 27695 USA; 2 Department of Food, Bioprocessing and Nutrition Sciences, North Carolina State University, Raleigh, NC 27695 USA; 3 Present Address: Department of Food Science, University of Massachusetts, Amherst, MA 01003 USA; 4 Present Address: Department of Biology, Dean of Faculty, United States Air Force Academy, CO 80840 USA; 5 Present Address: Department of Food, Bioprocessing and Nutrition Sciences, North Carolina State University, Raleigh, NC 27695 USA

**Keywords:** Norovirus–bacteria interactions, Glycoprotein, HBGA, Lectin, Enteric

## Abstract

**Objective:**

Research suggests human norovirus binding to histo-blood group antigen (HBGA)-like molecules on enteric bacteria may enhance viral pathogenesis; however, the properties of these bacterial ligands are not well known. Previous work identified, but did not characterize, seven norovirus-binding bacteria. To further examine this bacteria–virus binding interaction, enteric bacteria were analyzed via Western blot with anti-HBGA antibodies and lectins targeting HBGA-associated sugar components. Virus overlay assays using capsids from six different human norovirus strains further identified responsible ligands and strain dependent binding properties.

**Results:**

Each bacterial species possessed varying degrees of HBGA-like activity, and lectin binding further elucidated potential sugar residues involved (*N*-acetyl-galactosamine, α-d-galactose or α-l-fucose). Both GI and GII norovirus capsids bound specific bacterial ligand sizes, and generally corresponded to anti-HBGA Western blot patterns. A 35-kDa band reacted with all HBGA antibodies, bound all six of the noroviruses tested, and had a high affinity for the lectins. Collectively, this work characterizes the varying carbohydrate residues potentially responsible for norovirus–bacteria interactions and provides a basis for future ligand identification.

## Introduction

Human norovirus is the leading cause of viral gastroenteritis worldwide, yet there are still multiple questions related to its pathogenicity [1]. Researchers identified a putative cellular receptor for human norovirus—histo-blood group antigens (HBGAs)—the polymorphic terminal glycans found on red blood cells, gastrointestinal cells and secreted in saliva among other tissues and organs. Subsequent studies demonstrated the underlying complexities of the HBGA–norovirus relationship [2].

HBGA-like moieties are found in other animals, plants, and bacteria, but the potential relevance to human norovirus has only been recently explored. Initial studies demonstrated various human norovirus virus-like particles (VLPs) bind to the exopolysaccharide layer of *Enterobacter cloacae*, prompting the development of a putative cell culture model capable of productive human norovirus replication in the presence of either synthetic HBGAs or heat-killed *E. cloacae.* This work suggests luminal HBGAs and a bacterial component may work in tandem during a norovirus infection, while additional studies focused on identifying other bacteria capable of similar interactions [3–5].

While these studies collectively suggest versatility of the norovirus–bacteria interaction, the selected bacteria make up a small portion of the thousands of species present in the human gastrointestinal tract. The findings reported here build upon a recent study [6] on a larger, more diverse set of bacterial species representative of the human gut. The purpose of this study was to further characterize the nature of human norovirus–bacteria binding and identify a basis for future bacterial ligand identification.

## Main text

### Materials and methods

Virus-like particles (VLPs) and antibodies (Ab3912 and NS14) were provided courtesy of Dr. Robert Atmar (Baylor College of Medicine, Houston, TX, USA). Abcam antibodies corresponded to blood groups AB (ab24223), B (ab24224), H (ab24213), Lewis a (ab2967), Lewis b (ab3968), and Lewis y (ab3359). Blood group A and Lewis x antibodies failed to interact with each respective positive control, and were omitted.

Reference strains *Staphylococcus aureus* (ATCC 25235) and *Enterobacter cloacae* (ATCC 13047), in addition to five previously isolated strains (*Klebsiella* spp., *Bacillus* spp., *Enterococcus faecium*, *Citrobacter* spp., and *Hafnia alvei*) [3] were used in this study. Bacteria were grown aerobically at 37 °C overnight in 40 ml of half-strength tryptic soy broth (TSB) [6].

Overnight cultures were centrifuged and resuspended in 4 ml of chilled 1× phosphate buffered saline (PBS; pH 7.2), then sonicated at an amplitude setting of 60% for 10 s, followed by 20 s on ice, for seven rounds. Sonicates were mixed 1:1 with Laemmli buffer (Bio-Rad Laboratories) and boiled for 5 min. 25 µl aliquots were loaded into 12% mini-PROTEAN TGX gels (Bio-Rad Laboratories) with a spectra multicolor broad range ladder (Thermo Fisher Scientific), then run at 200 V in a Tris–glycine buffer.

Western blots were performed at room temperature using HBGA primary antibodies and lectins. SDS-PAGE protein gels were transferred to 0.45 μm nitrocellulose membranes and blocked at 4 °C overnight in SuperBlock (Thermo Fisher Scientific). The membranes were incubated for 1 h with PBS containing 0.5% skim milk/0.05% Tween 20 and a 1:500 ratio of the appropriate primary antibody. Membranes were washed thrice in PBS-0.5% Tween (PBS-T), then exposed to secondary antibody (Anti-mouse IgG-alkaline phosphatase; Sigma-Aldrich) diluted 1:5000 in PBS with 5% skim milk-0.5% Tween for 2 h. Membranes were washed and developed with BCIP/NBT solution (MP Biomedicals). Biotinylated HBGAs (A, B or H; Glycotech) were included as positive controls, while growth media, previously shown to contribute to blood group activity [7], was included as a negative control.

Lectins are highly specific sugar binding proteins. To determine if individual sugars could be linked to norovirus binding, a Western protocol utilized 10 μg of biotinylated lectins isolated from: *Bandeiraea simplicifolia* (Sigma-Aldrich), *Dolichos biflorus* (Bio-world), and *Ulex europaeus agglutinin* (Vector Laboratories) in place of primary antibody; and streptavidin-conjugated horseradish peroxidase (Invitrogen) at a 1:5000 dilution followed by addition of TMB-Blotting Substrate Solution (Thermo Fisher) for signal development. HBGAs containing (positive) or missing (negative) the sugar of interest were used as controls.

Bacterial binding of anti-HBGA antibodies is only relevant to a potential infection model if these same residues also bind to norovirus. To elucidate this relationship, a virus overlay protocol was adapted from Kikkert et al. [8]. The nitrocellulose membrane was washed in binding buffer (25 mM Tris–HCl [pH 7.5], 50 mM NaCl, 2 mM dithiothreitol [DTT], 2 mM EDTA, 0.25% Tween 20), then washed four times in renaturation buffer (25 mM Tris–HCl [pH 7.5], 50 mM NaCl, 2 mM DTT, 2 mM EDTA), and incubated overnight. The blot was washed twice in 5% skim milk-0.05% Tween 20, followed by a 30 min incubation in overlay buffer (5% skim milk-0.05% Tween 20, 2% polyvinylpyrrolidone). Diluted VLPs (2 μg/ml) were added to the overlay buffer, incubated with the blot for 2 h, washed as before and exposed to primary antibody [Ab3912 (GI.1, GI.6, GI.7) and NS14 (GII.1, GII.4 Sydney, GII.4 Grimsby, GII.17)] and developed as described. As a positive control, 10 μl of 1:1000 diluted norovirus antibody was included in each blot for viral adherence. Growth media was included as a negative control. An overlay was completed with the norovirus surrogate MS-2 as an additional negative control.

### Results and discussion

**Fig. 1:**
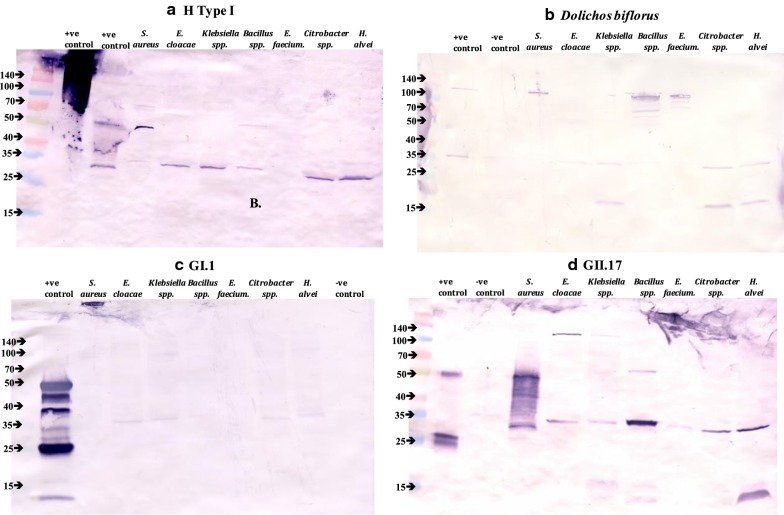
Representative Western Blots, **a** anti-H Type 1 antigen; **b** lectin *Dolichos biflorus*; **c** GI.1 Norwalk; and **d** GII.17. The numbers and arrows on the left of each blot correspond with the protein size in kDa

These results are consistent with early studies documenting that HBGA-like molecules occur commonly in bacterial species [7] and are consistent with more recent studies [9–11] suggesting HBGA-like activity differs by bacterial strain. The observed HBGA activity was unique to each bacterium based on (1) the number of anti-HBGA reactive bands; and (2) the molecular weight (ranging from 15- to 140-kDa) of each reactive band (Table 1, Additional file 1). All bacteria tested interacted with at least two different anti-HBGA antibodies, although the intensity and size of each band differs (Table 1; Fig. 1a). Each bacterium tested possessed its own HBGA profile, with similarities observed between the Gram-negative *Enterobacteriaceae* tested; however, no discernable binding pattern emerged amongst the Gram-positives. This observation supports previous research highlighting Gram-negative and Gram-positive bacteria interacting with norovirus using different mechanisms, as VLPs were found in association with the outer membrane of both *S. aureus* and *E. faecium*, while preferentially binding to protruding structures in Gram-negative bacteria [6]. Despite the differences observed, a 35-kDa band was present in six of the seven bacteria tested, and all of the anti-HBGA banding patterns. The outlier, *E. faecium*, possessed the overall lowest degree of HBGA-like activity (Table 1; Fig. 1a, Additional file 1).

**Table 1:** Bacterial HBGA and lectin binding activity with corresponding Western blot band size

HBGA/Lectin	H/*Ulex europaeus* (α-l-fucose: H type 2)								AB/*Dolichos biflorus* (GalNAc: A type 1)							
Protein size (kDa)	140	100	70	50	35	25	17	15	140	100	70	50	35	25	17	15
*S. aureus*	−/+			+/−	+/−				+/+	+/−	+/−		+/−			
*E. cloacae*					+/+				+/−			−/+	+/+		−/+	
*Klebsiella* spp.					+/+		−/+		+/−				+/+		−/+	
*Bacillus* spp.	−/+	−/+		+/−	+/+				+/+	+/+	+/−		+/+			
*E. faecium*	−/+								+/+	+/−			−/+			
*Citrobacter* spp.				+/+	+/+		−/+		+/−	+/−			+/+		−/+	
*H. alvei*				+/−	+/+		−/+		+/−	+/−			+/+			

HBGA/Lectin	B/*Bandeiraea simplicifolia* (α -d-Gal: B)							
Protein size (kDa)	140	100	70	50	35	25	17	15
*S. aureus*	+/+				+/−	+/−		
*E. cloacae*	+/−				+/−	+/−	−/+	
*Klebsiella* spp.	+/−				+/−		−/+	
*Bacillus* spp.	+/+	−/+			+/−			
*E. faecium*	+/+							
*Citrobacter* spp.	+/−				+/−	+/−	−/+	+/−
*H. alvei*	+/−				+/−		+/+	+/−

+ Indicates band observed at corresponding kDa and − indicates no band observed in three replicates. Each HBGA result (left side of each column) is coupled with the specific lectin sugar targeted from that residue (right side of each column)

Lectin binding assays attempted to (1) map the specific sugar residues present in the bacteria [i.e. *N*-acetyl-galactosamine (*Dolichos biflorus*), α-d-galactose (*Bandeiraea simplicifolia*), and α-l-fucose (*Ulex europaeus*)]; and (2) serve as an alternative to anti-HBGA antibodies, as additional motifs may be responsible for norovirus binding activity in bacteria. Of the four core sugars associated with human histo-blood group antigens (i.e. fucose, galactose, galactosamine and glucosamine), a combination of two of the four are required for HBGA activity [7]. Potentially, combinations uncommon among human HBGAs may be present on bacterial glycoproteins, as evidenced by the VLPs binding to some bacterial residues that were not bound by the anti-HBGA antibodies (Tables 1, 2; Fig. 1b; Additional file 1). These data suggest norovirus–bacteria interactions may be sugar specific and target individual glycan structures in lieu of the larger human HBGA complexes previously identified [3].

**Table 2:** VLP binding and corresponding Western blot band size

Norovirus VLP	GI.1					GI.6					GI.7				
Protein size (kDa)	140	100	50	35	17	50	35	25	17	15	140	100	50	35	25
*S. aureus*	+	+	+	−	−	+	+	+	−	−	−	−	+	–	−
*E. cloacae*	−	+	−	+	−	−	+	−	+	−	+	+	−	+	−
*Klebsiella* spp.	+	+	+	−	−	−	+	−	−	−	+	+	−	+	−
*Bacillus* spp.	+	+	−	+	−	−	+	+	+	−	+	+	−	+	−
*E. faecium*	−	+	−	−	−	−	–	+	−	−	+	−	−	−	−
*Citrobacter* spp.	−	+	−	+	−	−	+	−	+	−	+	+	−	+	−
*H. alvei*	+	+	+	+	+	−	+	−	+	+	+	+	−	+	+

Norovirus VLP	GII.4 Sydney					GII.17				
Protein size (kDa)	140	50	35	25	17	140	50	35	17	15
*S. aureus*	+	+	+	−	−	−	−	+	−	−
*E. cloacae*	+	+	+	−	−	+	−	+	−	−
*Klebsiella* spp.	+	+	+	−	−	+	−	+	+	−
*Bacillus* spp.	−	+	+	+	+	−	+	+	+	+
*E. faecium*	−	+	+	−	−	−	−	+	−	−
*Citrobacter* spp.	+	+	+	−	−	+	−	+	−	−
*H. alvei*	+	+	+	−	−	−	−	+	−	+

+ Indicates band observed at corresponding kDa and − indicates no band observed in two replicates

To determine which bands may be relevant for norovirus binding (Table 1; Fig. 1a, b), virus overlays were completed using three GI VLPs (corresponding to GI.1, GI.6, and GI.7 strains) and three GII VLPs (corresponding to GII.4 Sydney, GII.4 Grimsby and GII.17 strains). The binding patterns for the VLPs are shown in Table 2, while representative GI and GII Western overlays may be seen in Fig. 1c, d, respectively. Comprehensive Western blot results may be seen in Additional file 1. Overall, bacterial elements bound all VLPs tested, and the overlay results mostly corresponded to bands with HBGA activity. The 35-kDa bands on Gram-negative bacteria, which possessed the highest degree of both HBGA-like and lectin activity, bound all norovirus VLPs tested. For the Gram-positive bacteria, *Bacillus* spp. maintained a consistent binding profile across all VLPs tested, with a 35-kDa band, while *S. aureus* displayed binding at a 50-kDa band for all of the GI VLPs tested, and a 35-kDa band across GIIs. Conversely, there was no discernible GI VLP binding pattern for *E. faecium*, as it bound to 100-kDa (GI.1), 25-kDa (GI.6) and 140-kDa (GI.7) residues. There are two compelling trends from this data. Interestingly, the bacteria with limited HBGA activity still bound the norovirus VLPs [12], and preliminary work (GII.1) suggests a possible bacteria–virus binding for norovirus strains with no known human HBGA ligand. Given this observation, there may be an important structural difference between human and bacterial HBGA-like moieties. Additionally, some of the VLPs bound to residues for which there was no previously identified HBGA-like activity (Tables 1, 2). This phenomenon was mainly observed for lower molecular weight bands, but may also explain the strong reactions observed of norovirus binding to *E. faecium*, despite the poor performance observed with both HBGA and lectin binding. There are several possible reasons for this observation. The antibodies used in this study were raised against human HBGAs, and it is possible that human HBGAs are structurally different from their bacterial counterparts, resulting in reduced antibody binding for the bacterial moieties. Alternatively, the glycoproteins associated with the small bands or with the previously unobserved bands may not have been abundant enough to yield a discernable signal using a more broadly reactive HBGA antibody, but the high concentration of the VLPs facilitated binding. It may also be due to differences between the nature of the antibody or the VLP binding to the glycan.

## Conclusions

This study characterizes the interactions between gut-associated bacteria and human norovirus, suggesting both Gram-negative and Gram-positive bacteria possess HBGA-like moieties or closely related sugars. These bacterial components are capable of binding both GI and GII human norovirus; although the size of the glycoprotein, the overall HBGA-activity and number of viruses bound by each bacterial residue vary. This work lays the foundation for determining the potential role of gut microbiota in the human norovirus infection cycle and for identifying specific glycoproteins responsible for binding human norovirus.

## Limitations

- Reactivity of the Western blot antibodies. The antibodies were made against human HBGA antigens, leaving a potential for cross reactivity and sometimes ambiguous results.
- Western blot negative bacterial control. Bacteria examined included *Escherichia coli* DH5α, *Staphylococcus epidermidis* ATCC 35984, *Pantoea agglomerans*, *Pantoea ananas*, and *Pseudomonas moraviensis*, which have been described as either not possessing HBGA-like moieties (*S. epidermidis* [9]), or minimally interacting with human norovirus (*P. agglomerans*, *P. ananas*, and *P. moraviensis* [13]). All of the bacterial species tested interacted with the anti-HBGA antibodies. It is possible that some bacterial proteins (like *Staphylococcus aureus* protein A [14]) could specifically bind antibodies beyond a glycan interaction; however, this was not observed using the MS2 control (no bands) and bands at the sizes of these proteins were generally not observed. Previous work shows bacteria only required two sugars instead of a complete human antigen for positive blood group activity [7], which may explain the ubiquitous nature of bacterial HBGAs observed in this study. As outlined below, further controls were applied to confirm the role of bacterial sugars in HBGA activity.
- Western blot positive control. Synthetic biotinylated HBGAs, did not migrate well in the SDS-PAGE protein gel, and showed variability from batch to batch (Fig. 1a, column 2).
- HBGA activity confirmation. Several approaches knocked down binding to HBGA-like compounds, through modification of the terminal sugar residue or competition. To modify the sugar residues, three strategies were attempted: treatment with sodium periodate (100 mM) to oxidize carbohydrates [15]; treatment with sulfo-NHS-acetate (100uM) to block amine groups [16]; and digestion with *Vibrio cholera* neuraminidases to cleave sialic acid [17] (Additional file 1). Of these, the sodium periodate and sulfo-NHS-acetate reduced binding, while the neuraminidase treatment did not. To further pinpoint implicated sugars, HBGA Western blots were blocked with different potentially competitive sugars: lactose, *N*-acetyl galactosamine and N-acetyl lactosamine. Of these sugars, only *N*-acetyl lactosamine had an effect on signal (Additional file 1).

## Supplementary information

**Additional file 1: Figure S1.** Western blots using antibodies against **A** Le^a^, **B** Le^b^ and **C** Le^y^ antigens. **Figure S2.** Representative blots. **A**–**C** Western blots with **A** anti-H Type 1, **B** anti-AB, **C** anti-B antibodies; **D**, **E** lectin overlay blots; and **F**–**I** norovirus VLP overlay blots. **Figure S3.** Treated GII.17 overlay Western blots. **A**, **B** Sodium periodate treatment, **A** control and **B** treated; **C**, **D** Sulfo-NHS-acetate treated, **C** control and **D** treated; **E**–**H** sugar competition, **E** control, **F** lactose, **G** *N*-acetyl lactose and **H** *N*-acetyl galactose.

## Data Availability

All data is available in the provided additional files.

## References

[1] Newman KL, Leon JS (2015). Norovirus immunology: of mice and mechanisms. Eur J Immunol.

[2] Tan M, Jiang X (2005). Norovirus and its histo-blood group antigen receptors: an answer to a historical puzzle. Trends Microbiol.

[3] Almand EA, Moore MD, Jaykus L-A (2017). Norovirus binding to ligands beyond histo-blood group antigens. Front Microbiol.

[4] Bartnicki E, Cunha JB, Kolawole AO, Wobus CE. Recent advances in understanding noroviruses. F1000Research. 2017;6. 10.12688/f1000research.10081.1 PMC5270584 28163914

[5] Karst SM (2016). The influence of commensal bacteria on infection with enteric viruses. Nat Rev Microbiol.

[6] Almand EA, Moore MD, Outlaw J, Jaykus L-A (2017). Human norovirus binding to select bacteria representative of the human gut microbiota. PLoS ONE.

[7] Springer GF (1961). Blood group activity of gram-negative bacteria. J Exp Med.

[8] Kikkert M, Meurs C, van de Wetering F, Dorfmüller S, Peters D, Kormelink R (1998). Binding of tomato spotted wilt virus to a 94-kDa thrips protein. Phytopathology.

[9] Miura T, Sano D, Suenaga A, Yoshimura T, Fuzawa M, Nakagomi T (2013). histo-blood group antigen-like substances of human enteric bacteria as specific adsorbents for human noroviruses. J Virol.

[10] Amarasiri M, Hashiba S, Miura T, Nakagomi T, Nakagomi O, Ishii S (2016). Bacterial histo-blood group antigens contributing to genotype-dependent removal of human noroviruses with a microfiltration membrane. Water Res.

[11] Li D, Breiman A, le Pendu J, Uyttendaele M (2015). Binding to histo-blood group antigen-expressing bacteria protects human norovirus from acute heat stress. Front Microbiol.

[12] Han L, Tan M, Xia M, Kitova EN, Jiang X, Klassen JS (2014). Gangliosides are ligands for human noroviruses. J Am Chem Soc.

[13] Baugher JL. Characterizing human norovirus binding to fresh strawberries. North Carolina State University. 2016.

[14] Falugi F, Kim HK, Missiakas DM, Schneewind O (2013). Role of protein A in the evasion of host adaptive immune responses by *Staphylococcus aureus*. MBio.

[15] Sudalai A, Khenkin A, Neumann R (2015). Sodium periodate mediated oxidative transformations in organic synthesis. Org Biomol Chem.

[16] Mattson G, Conklin E, Desai S, Nielander G, Savage MD, Morgensen S (1993). A practical approach to crosslinking. Mol Biol Rep.

[17] Moustafa I, Connaris H, Taylor M, Zaitsev V, Wilson JC, Kiefel MJ, Von Itzstein M, Taylor G (2004). Sialic acid recognition by *Vibrio cholerae* neuraminidase. J Biol Chem.

