# Supplementary material for: Characterization of human norovirus binding to gut-associated bacterial ligands
Source: BMC Res Notes. 2019 Sep 23;12:607. doi: 10.1186/s13104-019-4669-2 (PMC6755701; doi:10.1186/s13104-019-4669-2)
Supplement: Supplementary file 1 — Additional file 1: Figure S1. Western blots using antibodies against A Lea, B Leb and C Ley antigens. Figure S2. Representative blots. A–C Western blots with A anti-H Type 1, B anti-AB, C anti-B antibodies; D, E lectin overlay blots; and F–I norovirus VLP overlay blots. Figure S3. Treated GII.17 overlay Western blots. A, B Sodium periodate treatment, A control and B treated; C, D Sulfo-NHS-acetate treated, C control and D treated; E–H sugar competition, E control, F lactose, G N-acetyl lactose and H N-acetyl galactose. [file 13104_2019_4669_MOESM1_ESM.docx]

**Additional File 1.**

**100🡺**

**140🡺**

**50🡺**

**200🡺**

**70🡺**

**40🡺**

**35🡺**

**25🡺**

**15🡺**

C.

**100🡺**

**140🡺**

**50🡺**

**200🡺**

**70🡺**

**40🡺**

**35🡺**

**25🡺**

**15🡺**

B.

**100🡺**

**140🡺**

**50🡺**

**200🡺**

**70🡺**

**40🡺**

**35🡺**

**25🡺**

**15🡺**

A.

Figure S1. Western blots using antibodies against A) Le^a^, B) Le^b^ and C) Le^y^ antigens. Lane 1: ladder (kDa), lane 2: +ve control, lane 3: *S. aureus,* lane 4: *S. aureus,* lane 5: *E. cloacae,* lane 6: *Bacillus* spp., lane 7: *Citrobacter* spp., lane 8: *E. faecium,* lane 9: *Klebsiella* spp., and lane 10: *H. alvei*

**100🡺**

**140🡺**

**50🡺**

**70🡺**

**40🡺**

**35🡺**

**25🡺**

**15🡺**

B.

**100🡺**

**140🡺**

**50🡺**

**70🡺**

**40🡺**

**35🡺**

**25🡺**

**15🡺**

A. H Type I

**100🡺**

**140🡺**

**50🡺**

**70🡺**

**40🡺**

**35🡺**

**25🡺**

**15🡺**

C. B Antigen

**100🡺**

**140🡺**

**50🡺**

**70🡺**

**40🡺**

**35🡺**

**25🡺**

**15🡺**

E. *Dolichos biflorus*

**100🡺**

**140🡺**

**50🡺**

**70🡺**

**40🡺**

**35🡺**

**25🡺**

**15🡺**

D. *Ulex europaeus*

**100🡺**

**140🡺**

**50🡺**

**70🡺**

**40🡺**

**35🡺**

**25🡺**

**15🡺**

F. GI.6

**100🡺**

**140🡺**

**50🡺**

**70🡺**

**40🡺**

**35🡺**

**25🡺**

**15🡺**

I. GII.4 Grimsby

**100🡺**

**140🡺**

**50🡺**

**70🡺**

**40🡺**

**35🡺**

**25🡺**

**15🡺**

H. GII.4 Sydney

**100🡺**

**140🡺**

**50🡺**

**70🡺**

**40🡺**

**35🡺**

**25🡺**

**15🡺**

G. GI.7

Figure S2. Representative Blots. A-C, Western blots with A) anti-H Type 1, B) anti-AB, C) anti-B Antibodies, lane 1: ladder, lane 2: pos control (biotinylated H Type 1, A or B), lane 3: *S. aureus*, lane 4: *S. aureus* control strain*,* lane 5: *E. cloacae,* lane 6: *Klebsiella* spp., lane 7: *Bacillus* spp., lane 8: *E. faecium*, lane 9: *Citrobacter* spp., lane 10: *H. alvei*; D-E, Lectin blots with D) *Ulex europaeus* lane 1: ladder; lane 2: pos control (biotinylated H type 1 HBGAs), lane 3: *S. aureus*, lane 4: *E. cloacae*, lane 5: *Klebsiella* spp., lane 6 *Bacillus* spp.; lane 7: *E. faecium*, lane 8: *Citrobacter* spp., lane 9: *H. alvei* , lane 10: neg control E) *Dolichos biflorus*, lane 1: ladder; lane 2: pos control (biotinylated A HBGAs), lane 3: neg control, lane 4: *S. aureus*, lane 5: *E. cloacae*, lane 6: *Klebsiella* spp., lane 7 *Bacillus* spp.; lane 8: *E. faecium*, lane 9: *Citrobacter* spp., lane 10: *H. alvei*; F-I, Norovirus VLP overlays with F) GI.6; G) GI.7; H) GII.4 Sydney and I) GII.4 Grimsby VLP. Blot F,H&I: lane 1: ladder; lane 2: pos control, lane 3: neg control (in H pos control), lane 4: *S. aureus*, lane 5: *E. cloacae*, lane 6: *Klebsiella* spp., lane 7 *Bacillus* spp.; lane 8: *E. faecium*, lane 9: *Citrobacter* spp., lane 10: *H. alvei*; Blot G: lane 1: ladder; lane 2: pos control, lane 3: *S. aureus*, lane 4: *E. cloacae*, lane 5: *Klebsiella* spp., lane 6: *Bacillus* spp.; lane 7: *E. faecium*, lane 8: *Citrobacter* spp., lane 9: *H. alvei* and lane 10: neg control.A-E: three replicates, F-I: two replicates.

A.

B.

**100🡺**

**140🡺**

**50🡺**

**200🡺**

**70🡺**

**40🡺**

**35🡺**

**25🡺**

**15🡺**

**100🡺**

**140🡺**

**50🡺**

**200🡺**

**70🡺**

**40🡺**

**35🡺**

**25🡺**

**15🡺**

D.

C.

**100🡺**

**140🡺**

**50🡺**

**200🡺**

**70🡺**

**40🡺**

**35🡺**

**25🡺**

**15🡺**

**100🡺**

**140🡺**

**50🡺**

**200🡺**

**70🡺**

**40🡺**

**35🡺**

**25🡺**

**15🡺**

H.

G.

F.

E.

**100🡺**

**140🡺**

**50🡺**

**200🡺**

**70🡺**

**40🡺**

**35🡺**

**25🡺**

**15🡺**

**100🡺**

**140🡺**

**50🡺**

**200🡺**

**70🡺**

**40🡺**

**35🡺**

**25🡺**

**15🡺**

**100🡺**

**140🡺**

**50🡺**

**200🡺**

**70🡺**

**40🡺**

**35🡺**

**25🡺**

**15🡺**

**100🡺**

**140🡺**

**50🡺**

**200🡺**

**70🡺**

**40🡺**

**35🡺**

**25🡺**

**15🡺**

Figure S3. Treated GII.17 Overlay Western blots. A-B Sodium periodate treatment, A) control and B) treated; C-D Sulfo-NHS-acetate treated, C) control and D) treated; E-H sugar competition, E) control, F) lactose, G) N-acetyl lactose and H) N-acetyl galactose. Lane 1: ladder, lane 2: +ve, lane 3: *S. aureus,* lane 4: *E. cloacae,* lane 5: *Bacillus* spp., lane 6: *Citrobacter* spp., lane 7: *E. faecium,* lane 8: *Klebsiella* spp., lane 9: *H. alvei* and lane 10: -ve.
